# Editorial: Advanced processing technology for improving the nutritional profile of food

**DOI:** 10.3389/fnut.2023.1271312

**Published:** 2023-09-04

**Authors:** Dimas Rahadian Aji Muhammad, Danar Praseptiangga, Muhammad Zukhrufuz Zaman

**Affiliations:** Department of Food Science and Technology, Universitas Sebelas Maret, Surakarta, Indonesia

**Keywords:** chitin, cinnamon essential oil (CEO), yeast fermentation, breadfruit, maize

Nutritional profile and bioactive compound content of food have gained high interest nowadays, both by the food industry as well as the consumer following the recent trend of healthy food consumption. Therefore, efforts have been made by scientists to advance processing methods aiming to either prevent the degradation or even improve the nutritional profile and bioactive compound content of the food. This is based on the fact that processing has a significant impact on the characteristic of foods, including its nutritional and bioactive compound content. To fulfill the consumer's need on the healthy food, knowledge and information related to nutritional profile of food as well as the influence of different processing techniques on the changes that occur in nutritional content and bioactive component of foods are substantial. More than that, food processing focusing on the improvement of the nutritional and bio-active compound content of food, including enrichment and fortification must be done. Recently, extensive researches have been carried out on the characterization of various materials containing micro- and macro-nutrients as well as bio-active compounds. Also, impact of processing on the nutritional content and bio-active compounds of food, new product development and fortified foods as well as new processing techniques for improving and/or preserving the nutritional content and bio-active compounds within food have been studied.

As such, the character of breadfruit (*Artocarpus Altilis*) along with its processing technique as well as its food industry application has been reviewed by Mehta et al.. As well-known, flour and starch are the main products of breadfruit which are potentially used for the formulation of gluten-free products. Washing, peeling, slicing, blanching, drying and milling are the main processing step of breadfruit flour making. Breadfruit flour can be further processed into starch by blending the flour with water, followed by precipitation, centrifugation and drying. The flour and starch can be used for different application in the food products including baked goods, carbohydrate staples such as pasta and noodle, beverages and dairy products as well as meat analogs for improving the nutritional properties of the products as breadfruit is a good source of carbohydrate, dietary fiber, protein, micronutrients and carotenoid antioxidants. Bai et al. studied the effect of chitin and crayfish shell fortification on the physicochemical property and starch digestion of puffed biscuits made from wheat flour and glutinous rice flour. This is an interesting innovation as chitin is a non-toxicity, biodegradability and biocompatibility polysaccharide while crayfish shell is a source of chitin alongside an antioxidant and a potential source of beneficial dietary fiber. This study successfully shown that chitin and crayfish shell inhibited starch digestion. The estimated glycemic index of the puffed biscuits enriched with chitin (15–20%) was below 55. This is a promising finding as the basis of further research particularly for designing fried puffed snacks for special segment such us consumer with chronic diseases including diabetes, cardiovascular disease, and obesity.

Zhang et al. discover an effective method by destroying the cell wall of *Sanghuangporus vaninii* aiming to extract its hypolipidemic polysaccharide. Extraction of active compound, including hypolipidemic polysaccharide, is crucial as the first step for food fortification. Some important parameters in the extraction process are processing temperature, processing time and the concentration of HCl. The extractable polysaccharide was proven to have lipid-lowering activity with significantly decreased lipid accumulation and levels of the triglyceride and total cholesterol in zebrafish larvae. Meanwhile, Tambo Tene et al. studied the effect of variety and malting conditions on proteolytic activity, free amino nitrogen and soluble protein contents of maize. They found that the maize malting condition, including soaking time, plant salt concentration, soaking temperature, sprouting time and ripening time significantly affected the proteolytic activity, free amino nitrogen content and soluble protein implying that optimal conditions must be selected by the food industry for gaining the targeted amount of free amino nitrogen and soluble protein content as well as proteolytic activity. The chosen flours can be applied in the formulation of various food products for the improving the nutritional properties of the food.

An example of the prevention of nutritional and quality degradation of food is shown by Wang et al.. In their research, cinnamon essential oil vapor was used to prevent chilling injury of “Feicheng” peach. It is clearly shown that vapor phase of cinnamon essential oil can play an important role in alleviating the quality deterioration in aroma and flesh color of “Feicheng” peaches caused by CI. This implies that the cinnamon essential oil vapor may be useful to delay the change in the nutritional profile of “Feicheng” peach during chilling storage. Improvement of nutritional of food by advanced processing technology is shown the study of Fu et al. They found that a traditional product from Japan called Angelica keiskei Jiaosu prepared by yeast fermentation contains bioactive compounds showing promising anti-obesity effects. This conclusion was based on the several parameters including serum levels of triglyceride, total cholesterol, leptin and glucose. This product, therefore, can be expected to serve as a potential anti-obesity functional food.

The six papers composing this Research Topic offer a new insight on the advanced processing technology for improving the nutritional profile as well as bioactive compounds of food. This may create a new paradigm for future researches.

## Author contributions

DM: Conceptualization, Funding acquisition, Project administration, Resources, Supervision, Writing—original draft. DP: Conceptualization, Funding acquisition, Validation, Writing—review and editing. MZ: Funding acquisition, Supervision, Validation, Writing—review and editing.

